# Overcome the Brightness and Jitter Noises in Video Inter-Frame Tampering Detection

**DOI:** 10.3390/s21123953

**Published:** 2021-06-08

**Authors:** Han Pu, Tianqiang Huang, Bin Weng, Feng Ye, Chenbin Zhao

**Affiliations:** 1 School of Mathematics and Information, Fujian Normal University, Fuzhou 350007, China; 20112009@bjtu.edu.cn (H.P.); fjhtq@fjnu.edu.cn (T.H.); yefeng@fjnu.edu.cn (F.Y.); 2 Digital Fujian Institute of Big Data Security Technology, Fujian Normal University, Fuzhou 350007, China; 3 Engineering Technology Research Center for Public Service Big Data Mining and Application of Fujian Province, Fujian Normal University, Fuzhou 350007, China; 4 School of Cyber Science and Engineering, Wuhan University, Wuhan 430072, China; 2020102210008@whu.edu.cn

**Keywords:** brightness-adaptive, robust optical flow algorithm, relax the brightness constancy assumption, texture changes fraction feature

## Abstract

Digital video forensics plays a vital role in judicial forensics, media reports, e-commerce, finance, and public security. Although many methods have been developed, there is currently no efficient solution to real-life videos with illumination noises and jitter noises. To solve this issue, we propose a detection method that adapts to brightness and jitter for video inter-frame forgery. For videos with severe brightness changes, we relax the brightness constancy constraint and adopt intensity normalization to propose a new optical flow algorithm. For videos with large jitter noises, we introduce motion entropy to detect the jitter and extract the stable feature of texture changes fraction for double-checking. Experimental results show that, compared with previous algorithms, the proposed method is more accurate and robust for videos with significant brightness variance or videos with heavy jitter on public benchmark datasets.

## 1. Introduction

The rapid development and spread of low-cost and easy-to-use video editing software, such as Adobe Premiere, Photoshop, and Lightworks, makes it easier to tamper with digital video without efforts. Inter-frame forgery happens quite often. It includes inserting frames into a video sequence or removing frames from a video sequence [1]. These tampered videos may be indistinguishable to the naked eye. Thus, they may harm judicial forensics, media reports, e-commerce, finance, and public security. Therefore, it is necessary to develop methods to help human eyes identify tampered videos [1].

A considerable amount of effort has been devoted to inter-frame forgery detection. Most of these approaches are based on the successful extraction of some characteristics of the video. For example, some recent works detected tampered video by calculating the optical flow between frames [2,3,4,5,6]. However, this process could be severely interrupted by illumination noises, which invalidates the extraction of optical flow features [7,8]. Besides, jitter noise may also affect correlation consistency between adjacent frames in the video [9,10], causing many false detections.

For the forgery detection of the videos with noises, a few methods have been developed, including low-rank theory for video with blur noise [11], the coarse-to-fine approach under the condition of regular attacks, including additive noise and filtering [12]. However, these works did not consider brightness and jitter noises. Videos with brightness changes and jitter videos are common in real life—e.g., most of the videos are shot by cell phones. Although the motion-adaptive method [13] considered both brightness and jitter noises, it was not suitable for the lowest motion video with minor changes between adjacent frames, which is quite popular. Moreover, these methods also do not consider or validate the effect of multi-tamper.

We propose a novel framework that not only takes into account both brightness and jitter noises, but also considers the lowest motion video. To deal with considerable illumination noise, we introduce the relaxing brightness constancy assumption [14] and develop a linear model to present the physical intensity change. To deal with subtle illumination noises, we introduce intensity normalization [15]. To deal with the false detection caused by video jitter, we propose motion entropy and stable texture changes fraction features of the video for double-checking. In addition, the improved robust optical flow is insensitive to the motion level of the video. Moreover, the texture changes fraction feature can also describe the subtle inter-frame differences of the lowest motion video. Therefore, our method is also suitable for the lowest motion video. Experimental results on three public video databases show that our method can be applied to the videos with brightness variance, the videos with significant jitter, and the lowest motion videos. Furthermore, our approach can not only locate the forgery precisely, but it can also estimate the way of multi-forgery on tampered positions.

The rest of this paper is organized as follows. In Section 2, we briefly introduce the related work for inter-frame forgery detection. In Section 3, we briefly describe the preliminaries in this paper. Section 4 describes the proposed scheme in detail. We provide the evaluation of optical flow computation in Section 5. Experimental results and analysis are presented in Section 6, and we draw a conclusion and discuss future works in Section 7.

## 2. Related Work

Most of the prior works detected forgeries based on the analysis of correlations between frames, which relies on features extracted from videos. As noises in videos could significantly affect feature extraction and correlation analysis, we classify the existing methods into two categories: methods without considering noises and methods considering noises.

### 2.1. Methods without Considering Noises

In terms of the type of features, previous methods could be divided into two categories: image-features-based and video-features-based. Methods in the first category usually extracted image features of each frame, such as texture features [9], color characteristics [9,16], histogram features [16], structural features [17], etc. Methods in the second category mainly utilized the impact of tampering on video features, including video encoding characteristic [18,19,20], double compression [21], motion features such as errors in motion estimation [10], optical flow, predict residual gradients [19], and brightness features such as segmented brightness variance descriptor (BBVD) [2], illumination information [4], etc.

Although these methods have been validated on videos from public data sets, they generally did not consider noises. They could probably generate incorrect results on real-life videos containing various noises. For example, the performance of methods [2,4,19] declines due to illumination noise, and the methods [9,18] are susceptible to jitter noises in real-life videos.

### 2.2. Methods Considering Noises

To address the issue of feature extraction in the blurry video, Lin et al. [11] adopted low-rank theory to deblur video, fusing multiple fuzzy kernels of keyframes by low-rank decomposition. Jia et al. [12] proposed a video copy-move detection method based on robust optical flow features. Furthermore, they also adopt adaptive or stable parameters to detect the tamper under the condition of regular attacks, including additive noise and filtering. The method [12] is limited or only validated by copy-move forgery. However, our proposed approach applies to all tampering operations, including frame insertion, frame copy-move, frame replication, and frame deletion. Feng et al. [13] adopted a frame deletion detection method based on the motion residuals feature. They embrace the postprocessing forensic tools, including the automatic color equalization (ACE) forensics and mean gradient evaluation, to eliminate the detection interference caused by illumination and jitter noises.

Illumination noises and jitter noises have side effects on the detection result. However, few works take both brightness and jitter noises into account at the same time. Although [13] has considered both noise factors, it does not fit the lowest motion strength video. While our work takes both brightness and jitter into account at the same time, it is also suitable for the lowest motion videos.

## 3. Preliminaries

### 3.1. Horn and Schunck (H&S) Method

When a moving object in the three-dimensional world is projected onto a two-dimensional plane, optical flow (OF) is the relative displacement of the pixels of the image pairs [8]. Specifically, the optical flow method uses the information difference between adjacent frames to describe the movement of objects in a three-dimensional world [7]. OF has been widely applied in various scenes, such as object segmentation, target tracking, and video stabilization [22].

Horn and Schunck (H&S) method [8] is a classical OF estimation algorithm, which is based on three major premise assumptions: brightness consistency, the spatial coherence of neighboring pixels, and small motion of the pixel [23]. Given a video sequence, the pixel intensity at the position (x,y)  of *t*-th frame is I(x,y,t) , the brightness consistency can be described by the Equation

(1) I(x+dx,y+dy,t+dt)=I(x,y,t)

where dx and dy correspond to the slight change of the movement over *dt*, then Equation (1) can be expanded by the first-order Taylor series

(2) $$I(x+dx,y+dy,t+dt)\approx I(x,y,t)+\frac{\partial I}{\partial x}dx+\frac{\partial I}{\partial y}dy+\frac{\partial I}{\partial t}dt$$

Let $I_{x}=\partial I/\partial x,\;I_{y}=\partial I/\partial y,\;I_{t}=\partial I/\partial t$, then $I_{x}$, $I_{y}$, $I_{t}$ represents the change rate of the grey value of the pixel along the *x*, *y*, and *t* directions, respectively. Combining Equations (1) and (2), we can get the Equation

(3) ∂I∂xdx+∂I∂ydy+∂I∂tdt=Ixdx+Iydy+Itdt=0

According to the definition of speed Equation u=dx/dt and v=dy/dt, we obtain

(4) $$I_{x}u+I_{y}v+I_{t}=0$$

Equation (4) is the OF constraint equation, then we constrain the OF calculation problem to the minimum optimization problem of Equation (5), $E_{d}$ is the sum of the errors under the brightness constancy constraint, and there are two unknown variables: *u* and *v*. An equation cannot determine a unique solution, so a new condition $E_{s}$ needs to be introduced. $E_{s}$ is the constraint condition for smooth changes of OF over the entire image [24], which is shown in the Equation (6).

(5) $$E_{d}=\iint_{}{\left(I_{x}u+I_{y}v+I_{t}\right)}^{2}dxdy$$

(6) $$E_{s}=\iint_{}\left(|\nabla u{|}^{2}+|\nabla v{|}^{2}\right)dxdy=\iint_{\Omega }\left[{\left(\frac{\partial u}{\partial x}\right)}^{2}+{\left(\frac{\partial u}{\partial y}\right)}^{2}+{\left(\frac{\partial v}{\partial x}\right)}^{2}+{\left(\frac{\partial v}{\partial y}\right)}^{2}\right]dxdy$$

where ∇ represents the gradient operator.

The H&S algorithm converts the OF solution to the minimum optimization problem, shown as the following Equation (7). Equation consists of a grayscale change factor $E_{d}$ and a smooth change factor $E_{s}$. The ideal OF value *E* is relatively small, so the corresponding values of the grayscale change of $E_{d}$ and the speed change $E_{s}$ are also small, which meets the assumption of constant brightness and small motion, respectively.

(7) $$E=E_{d}+\lambda E_{s}=\iint_{}\left[{\left(I_{x}u+I_{y}v+I_{t}\right)}^{2}+\lambda \left(|\nabla u{|}^{2}+|\nabla v{|}^{2}\right)\right]dxdy$$

where ∇ represents the gradient operator and λ represents the smooth factor.

### 3.2. Robust Optical Flow Algorithm against Brightness Changes

The above classical OF calculation is usually incorrect when the image sequence has significant brightness changes, which exist in most real-life videos. Therefore, the OF algorithm was enhanced by relaxing brightness consistency assumptions [14].

Gennert et al. [14] relaxed brightness consistency assumptions by the Equation

(8) I(x+dx,y+dy,t+dt)=S(x,y,t)I(x,y,t)+T(x,y,t)

where S(x,y,t) and T(x,y,t) are constraint parameters for space and time.

Combining Equations (2) and (8), we can obtain

(9) $$\frac{\partial I}{\partial x}dx+\frac{\partial I}{\partial y}dy+\frac{\partial I}{\partial t}dt=(S-1)I+T$$

Let $sc=\underset{dt\to 0}{\lim}(S-1)/dt$,$tc=\underset{dt\to 0}{\lim}T/dt$, we combine (9) and (3) obtain

(10) $$I_{x}u+I_{y}v+I_{t}-scI-tc=0$$

The enhanced OF is calculated by solving the extreme value problem described by Equation (11). Compared with Equation (7), the enhanced OF algorithm is more robust by considering the brightness change.

(11) $$\begin{array}{l} \min_{u,v,sc,tc}E=E_{d}+\lambda_{s}E_{s}+\lambda_{sc}E_{sc}+\lambda_{tc}E_{tc}\\ E_{d}=\iint_{\Omega }{\left(I_{x}u+I_{y}v+I_{t}-scI-tc\right)}^{2}dxdy\\ E_{s}=\iint_{\Omega }\left(|\nabla u{|}^{2}+|\nabla v{|}^{2}\right)dxdy\\ E_{sc}=\iint_{\Omega }|\nabla sc{|}^{2}dxdy\\ E_{tc}=\iint_{\Omega }|\nabla tc{|}^{2}dxdy \end{array}$$

where $\lambda_{s},\lambda_{sc},\lambda_{tc}$ are smoothing factor, spatial domain constraint parameter, and time-domain constraint parameter, respectively. $E_{d}$, $E_{sc}$ are grayscale change factor and smooth change factor, respectively. $E_{sc}$ and $E_{tc}$ are spatial and time-domain constraint parameters, respectively.

## 4. Method

**Figure 1:**
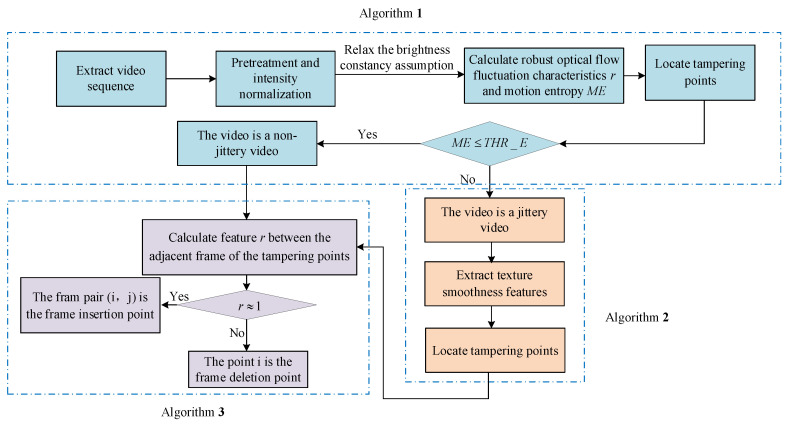
Detection process of the proposed framework.

We propose a novel framework to overcome the brightness and jitter noises in video inter-frame tampering detection. As illustrated in Figure 1, there are three algorithms in this framework. Firstly, Algorithm 1 reduces the impact of illumination changes in the input video sequence by the optical flow information. At the same time, if the motion entropy is more significant than a certain threshold, we detect jittery video by Algorithm 2. Based on the detected tampering points of the above two steps, Algorithm 3 makes the judgment of video tamper finally.

### 4.1. Algorithm 1: Reduce the Impact of Illumination Changes

The consistency of the OF has been proven to be an efficient tool to check the integrity of video [3,5,6]. Based on the enhanced OF algorithm described in the previous section, we design Algorithm 1 to reduce the impact of illumination changes; the main steps are shown as follows.

Step 1: Due to the brightness variations, the intensity of images should be normalized [15] before applying the optical flow method with a digital filter sequence. To cope with the high-frequency noise which affects the OF computation, we preprocess the input video by Gaussian filter [25].

Step 2: Based on the enhanced OF method described in Section 3 we extract the OF fluctuation feature $r_{i}$ to measure the similarity between adjacent frames of the video by Equation (12)

(12) ri=sum_OFiavg(sum_OFi)

$sum\_OF_{i}$ is the OF sum of the *i*-th video frame, which is calculated by Equation (13)

(13) $$\begin{array}{l} sum\_OF_{i}=\sum_{m=1}^{wid}\sum_{n=1}^{hei}(\left|u_{i}(m,n)\right|)+\left|v_{i}(m,n)\right|)\\ i=1,2,\ldots ,N-1 \end{array}$$

where wid,hei represent the width and height of the video frame, respectively. *N* is the video frames number.

The average OF sum $avg(sum\_OF_{i})$ in a sliding window centered on the i_th frame is calculated by the Equation:

(14) $$avg(sum\_OF_{i})=\{\begin{array}{cc} \frac{sum\_OF_{3}+sum\_OF_{4}}{2} & i=1\\ \frac{sum\_OF_{i-1}+sum\_OF_{i+1}}{2} & i\in (1,w+1)\cup (N-w,N)\\ \frac{\sum_{k=1}^{w}sum\_OF_{i+k}+sum\_OF_{i-k}}{2w} & i\in \left[w+1,N-w\right]\\ \frac{sum\_OF_{i-1}+sum\_OF_{i-2}}{2} & i=N \end{array}$$

where 2*w* is the width of the sliding window, $sum\_OF_{3}$ is the OF sum of the third video frame, $sum\_OF_{4}$ is the OF sum of the fourth video frame.

Step 3: Jitter frame pixels have small amplitude movements in the same motion direction [26], which has the consistency of motion direction. The video with consistent motion direction has small motion direction entropy. Therefore, we adopt motion direction entropy *ME* to perceive the consistency of video motion direction, which can sense video jitter.

*ME* can be calculated as follows: 1. Use the frame difference method [27] to calculate the binarized motion area. 2. Utilize *Shi-Tomasi* corner calculation method [28] to obtain the corner c(j) on the binarized motion area. 3. Combining the standard deviation S(θ) of the histogram of the OF direction, the motion entropy ME of the video is computed.

(15) $$ME_{i}=\left(\sum_{j\in c(j)}OF_{ij}\right)/S(\theta )$$

(16) $$\begin{array}{l} ME=std\left(ME_{i}\right)\\ i=1,2,\ldots ,N \end{array}$$

where $OF_{ij}$ is the OF of the corner c(j) in the i_th frame. The S(θ) is the standard deviation measure of the OF direction histogram, which measures the consistency of the direction histogram. $ME_{i}$ is the motion entropy of the i_th frame and std is the standard deviation of the N video frames.

Step 4: We judge whether the video is tampered with based on the continuity of the video frame feature sequence. THR_R is a threshold selected for the peak point of OF fluctuation feature sequence, and *C* is the variable counter for peak point. THR_E is the threshold selected for ME. If $r_{i}THR\_R$, the i_th frame is considered to be the suspected tamper point and C+=1 is used to count the number of suspected tamper points. When C≥1, it means the video has suspected tamper points. Under the premise of C≥1, if the detection result satisfies the condition ME≤THR_E, which means the video is not jittery. Then we can judge the video as a tampered video directly; if not, it indicates that the video is jittery. Therefore, the video needs to be further detected by Algorithm 2. The suspected tampered position detection process is summarized in Algorithm 1.
**Algorithm 1:** Reduce the impact of illumination changes$\mathrm{Input}:\;\mathrm{video}\;\mathrm{frames}I^{\left(p\right)}(1\leq p\leq N)$, set THR_R  as threshold selected for peak point,
            THR_R as threshold selected for ME.Output: store position of suspicious tampering point in *S.*1: S = ∅, *C* = 0 //*C* is the variable counter for peak point2: for i=1;i<N;i++ do
3:   calculate OF $\;\mathrm{fluctuation}\;\mathrm{feature}\;r_{i}$ and motion entropy ME$4:\;\;if\;r_{i}THR\_R$**then**5:      add iinto S6:      C+=17:  **end if**
8: **end for**
9: if C≥110:  if ME≤THR_E then11:   (a) return FORGED VIDEO
13:  (b) store S,C14:  **else** run Algorithm 2
15:  **end if**
16:**else return** ORIGINAL VIDEO
17:**end if**

### 4.2. Algorithm 2 Detects Jittery Video

**Figure 2:**
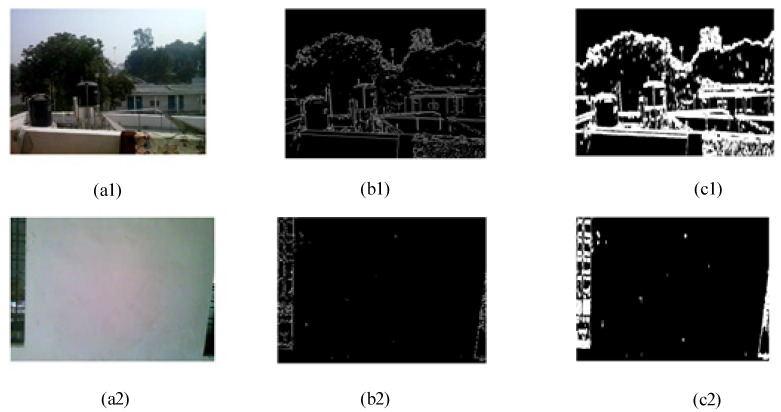
Textured area of video frames. (**a1**,**a2**) are the video frames; after compute the gradient structure information of (**a1**,**a2**), (**b1**,**b2**) are the corresponding binary mask; after perform morphological operations on (**b1**,**b2**), (**c1**,**c2**) are the textures area of video frames).

Video jitter refers to a small motion in the same motion direction of the video frame. Since the enhanced OF fluctuation feature r in Algorithm 1 is a global motion statistic, only using the feature r is likely to cause leak detection or false detection, especially in the case of severe video jitter. To eliminate the false negative detected point caused by video jitter, we adopt the video texture changes fraction TC to detect the jitter video. The TC feature captures the local details changes of different motion direction of the video frame, which is not captured the characteristics of the same motion direction of the jitter frame, so jittery frames are not identified as tampered frames. The TC feature is calculated by three steps:

Step 1: We compute the gradient structure information of the i_th frame as $\left\|\Delta I^{i}\right\|$. The corresponding binary mask $TM_{i}$ is obtained by the threshold $Th_{t}\;$for the gradient image $\left\|\Delta I^{i}\right\|$, and the binary mask of the video frame is shown as Figure 2b1,b2.

(17) $$\left\|\Delta I^{i}\right\|=\sqrt{{\left(I_{x}^{i}\right)}^{2}+{\left(I_{y}^{i}\right)}^{2}}$$

(18) $$TM_{i}=\{\begin{array}{ll} 255 & \left\|\Delta I^{i}\right\|Th_{t}\\ 0 & \left\|\Delta I^{i}\right\|\leq Th_{t} \end{array}$$

where $I_{x}^{i}$ is the partial derivative of the i_th frame in the *x*-direction, $I_{y}^{i}$ is the partial derivative of the i_th frame in the *y*-direction, and $\left\|\Delta I^{i}\right\|$ means the gradient structure information of the i_th frame.

Step 2: We perform morphological operations on the binary mask $TM_{i}$ to fill the gaps and remove small areas containing noise, as shown in Figure 2(c1,c2).

(19) $$TM_{i}=\left(TM_{i}•SE\right)\circ SE$$

where • means a closed operation of morphological operation, ∘ means an open operation of morphological operation, and SE is a structural element of open operation and closed operation.

**Figure 3:**
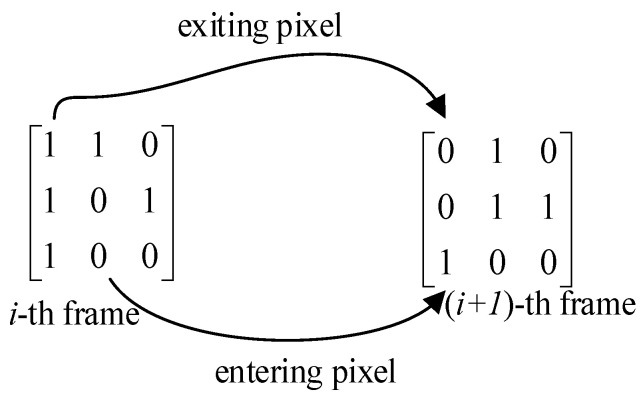
Statistics of video frame texture changes fraction.

Step 3: We calculate the texture changes fraction $TC(I^{i},I^{i+1})$ between $TM_{i}$ and $TM_{i+1}$ with Equation (20), and || is an absolute value operator. The value of 1 in i_th frame and 0 in (i+1)_th  frame is called the exiting pixel, shown by the arrow at the top of Figure 3, and its statistic is called Cout. On the contrary, the value of 0 in i_th frame and 1 in (i+1)_th frame is called entering pixels, shown by the arrow at the bottom of Figure 3, and its statistic is called Cin. The process of the detection algorithm based on video texture changes fraction TC is shown in Algorithm 2.

(20) $$TC(I^{i},I^{i+1})=\left|Cin_{i}-Cout_{i}\right|$$

Due to the picture continuity of video frames, the content similarity between adjacent frames is substantial, and the value TC is considerably small. If a certain number of frames are inserted or deleted, the video continuity will be destroyed. The larger the value TC, and the more likely the video is to be tampered.
**Algorithm 2:** Detection algorithm based on video texture changes fraction$\mathrm{Input}:\;\mathrm{video}\;\mathrm{frames}\;I^{\left(p\right)}(1\leq p\leq N)$, set THR_R1 as threshold selected for peak point Output: store position of suspicious tampering point in S1: S=∅,C=0 //Reset S,C in Algorithm 1
2: for i=1;i<N;i++ do
$3:\;\;\;\;\mathrm{calculate}\;\mathrm{video}\;\mathrm{texture}\;\mathrm{changes}\;\mathrm{fraction}\;TC(I^{i},I^{i+1})$$4:\;\;\;\;\;\;if\;TC(I^{i},I^{i+1})THR\_R1$ then
5:          add i into S6:          C += 17:    **end if**
8: **end for**
9: if C≥1 then
10:   (a) return FORGED VIDEO
11:   (b) store S,C12:**else** return ORIGINAL VIDEO
13:**end if**

### 4.3. Algorithm 3: Make the Judgement of Video Tamper

The exiting common video tamper operation can cause different tampering point on the extracted video feature sequence. More concretely, the deletion forgery causes a sudden peak in the feature sequence, and the insertion forgery causes two pikes. When $I^{i}$ is a frame forgery point and its previous frame is $I^{\left(i-1\right)}$. At the same time, $I^{j}$ is another frame forgery point, and its next frame is $I^{\left(j+1\right)}$. If $I^{\left(i-1\right)}$ and $I^{\left(j+1\right)}$ are very similar, then there is a video frame insert clip from $I^{i}$ to $I^{\left(j-i+1\right)}$. If not, the tamper detection method is a deletion forgery. The process of judgment of video tamper is shown in Algorithm 3.
**Algorithm 3:** judgment of video tamperInput:suspicious tampering point set in S, the variable counter for peak point C$\mathrm{Output}:\;\mathrm{frame}\;\mathrm{insertion}\;\mathrm{set}S_{insert\;}$$,\;\mathrm{frame}\;\mathrm{deletion}\;\mathrm{set}\;S_{delete}$ 
$1:\;S_{insert\;}=\emptyset ,S_{delete}=\emptyset$2: for i=1;i<C;i++ do
3:    for j=i+1;j<C;j++ do
4:      if j>C:
5:         add i$\;\mathrm{into}\;S_{delete}$6:      **else:**

7:           calculate  OF fluctuation feature r between frame S[i]−1and S[j]+18:         if r≈1:
9:              add i,j $\mathrm{into}\;S_{insert\;}$10:         **end if**
11:      **end if**
12:   **end for**
13: **end for**


## 5. Evaluation of Optical Flow Computation

### 5.1. Experimental Setup

**Figure 4:**
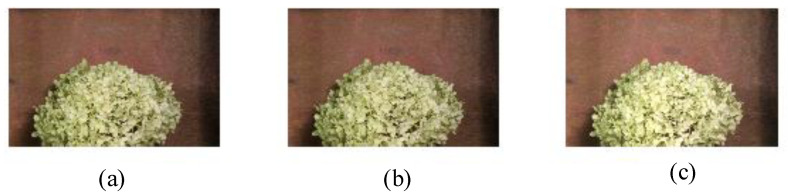
Hydrangea image pair. (**a**) frame10, (**b**) frame11, and (**c**) brightness change added on frame11 while M=1.1 and $C_{1}=10$.

To evaluate the enhanced OF algorithm of the proposed detection framework, we perform experiments on the benchmark dataset [29]. The dataset contains various image sequences and the corresponding ground-truth OF information, so we can quantify the robustness and accuracy of the enhanced OF algorithm. To evaluate the enhanced OF algorithm against dynamic brightness variation, the image *I* is multiplied by a factor *M*, and a constant $C_{1}$ is added to construct a model of dynamic brightness variation. The specific calculation process is shown in Equation (21). For example, Figure 4a,b show frame10 and frame11 of the Hydrangea sequence group in the dataset, respectively. When M=1.1 and $C_{1}=10$, Figure 4b is changed to Figure 4c. We need to calculate the OF information between Figure 4a,c.

(21) $$I=M*I+C_{1}$$

where $M\in [0.9,1.1],C_{1}\in [-10,10]$.

We estimate the OF information between Figure 4b and c, let $u_{i}^{gt}$,$v_{i}^{gt}$ represent the real OF information, and let $u_{i}^{e}$, $v_{i}^{e}$ represent the estimated OF information. We evaluate OF methods by two measures indicators: the average angular error (AAE) [30] and the end point error (EPE) [31]. The AAE and EPE are used to compare the difference between the ground truth OF and the estimated OF information. The smaller the values of AAE and EPE, the better the performance of the corresponding OF algorithm. We can also visually estimate OF algorithm performance by visualization of the flow map. Equation of AAE is shown in Equations (22) and (23).

(22) $$\phi (i)=\arccos\left[\frac{u_{i}^{gt}u_{i}^{e}+v_{i}^{gt}v_{i}^{e}+1}{\sqrt{{\left(u_{i}^{gt}\right)}^{2}+{\left(v_{i}^{gt}\right)}^{2}+1}\sqrt{{\left(u_{i}^{e}\right)}^{2}+{\left(v_{i}^{e}\right)}^{2}+1}}\right]$$

(23) $$\mathrm{AAE}=\frac{1}{N}\sum_{i=1}^{N}\phi (i)$$

### 5.2. Experimental Results and Analysis

**Figure 5:**
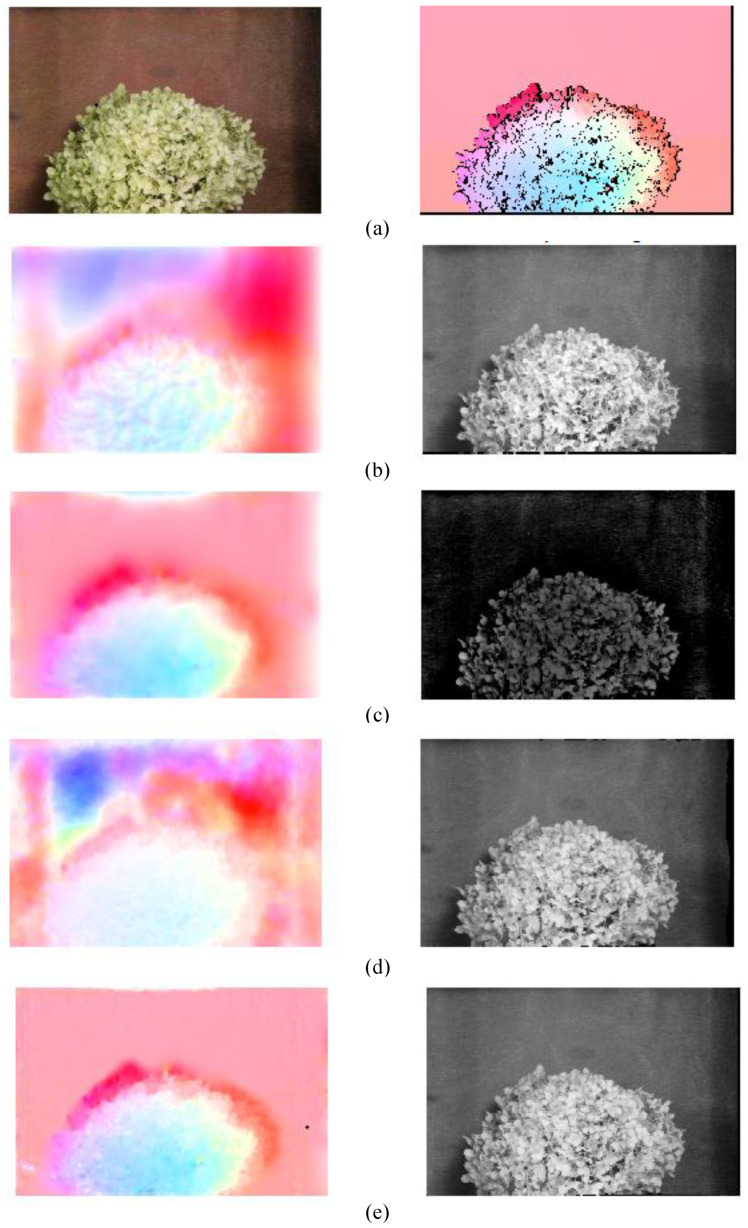
Comparison of different OF methods between frame10 and frame11 of Hydrangea images under brightness change (colored). (**a**) Original image and the ground-truth velocity field, OF and corresponding warped image of (**b**) ‘HS’, (**c**) ‘HS + IN’, (**d**) ‘HS + BR’, and (**e**) ‘the enhanced OF algorithm’.

The test results of different OF algorithms between frame10 Figure 4a and frame11 (Figure 4c under brightness change) in the Hydrangea sequence group are shown in Figure 5. The description of different approaches and parameter settings used for OF evaluation is shown in Table 1. The performance evaluation results of different OF algorithms are shown in Table 2. The original image and the ground-truth velocity field are shown in Figure 5a. The flow map and the warped image obtained by the HS algorithm are shown in Figure 5b. The flow map uses different colors and brightness to indicate the size and direction of the estimated OF, and the warped image represents frame11 warped to frame10 according to the estimated OF. At the same time, it is observed that the estimated flow map in Figure 5b and the ground truth in Figure 5a are significantly different. The error measures of AAE and EPE in Table 2 are also relatively large. It is observed that the HS algorithm is not suitable for the evaluation of the image sequence with dynamic brightness variation.

The evaluation result of the HS+IN (intensity normalization) algorithm is shown in Figure 5c. Compared to the HS algorithm, the values of AAE and EPE of the HS+IN algorithm are significantly reduced. The execution time is not much different, which indicates that the intensity normalization is beneficial to the OF calculation of image sequences with brightness changes.

The evaluation result of the HS+BR (brightness relaxing factor) algorithm is shown in Figure 5d. Compared to the HS algorithm, the values of AAE and EPE of the HS+BR algorithm is greatly increased, which indicates that just introducing the brightness relaxing factor is not beneficial to the OF calculation of image sequences with brightness changes.

Combining IN and BR, we propose the enhanced OF algorithm. The evaluation result is shown in Figure 5e, which is very close to the ground-truth velocity field in Figure 5a visually. The warped image is also similar to frame10. The values of AAE and EPE are small, which reaches single digits. The above indicators show that the enhanced OF algorithm proposed is suitable for the OF calculation of image sequence with brightness changes. We have made a trade-off between computational accuracy and time complexity.

## 6. Experimental Results and Analysis

We conduct extensive experiments in diverse and realistic forensic setups to evaluate the performance of the proposed detection framework in this section. The experimental data is introduced first. Then the setup of parameters and evaluation standards are suggested. Finally, we present the experimental results and comparison analysis with four existing state-of-art algorithms to detect accuracy and robustness.

### 6.1. Experimental Data

To evaluate the detection effect of the proposed method, we performed experiments on three public datasets, namely the SULFA Video Library (The Surrey University Library for Forensic Analysis) [32], the CDNET Video Library (a video database for testing change detection algorithms) [33], and the VFDD Video Library (Video Forgery Detection Database of South China University of Technology Version 1.0) [34], respectively. There are about 200 videos in total. The scenes in the video library are as follows:

(1) The video library includes videos of different motion levels, including slow motion, medium motion, and high motion.

(2) The video library contains videos of different brightness intensities and different scenes (indoors and outdoors).

(3) The video library includes a variety of mobile phone videos, as well as camera videos, which were taken with or without a tripod.

### 6.2. Experimental Setup

**Figure 6:**
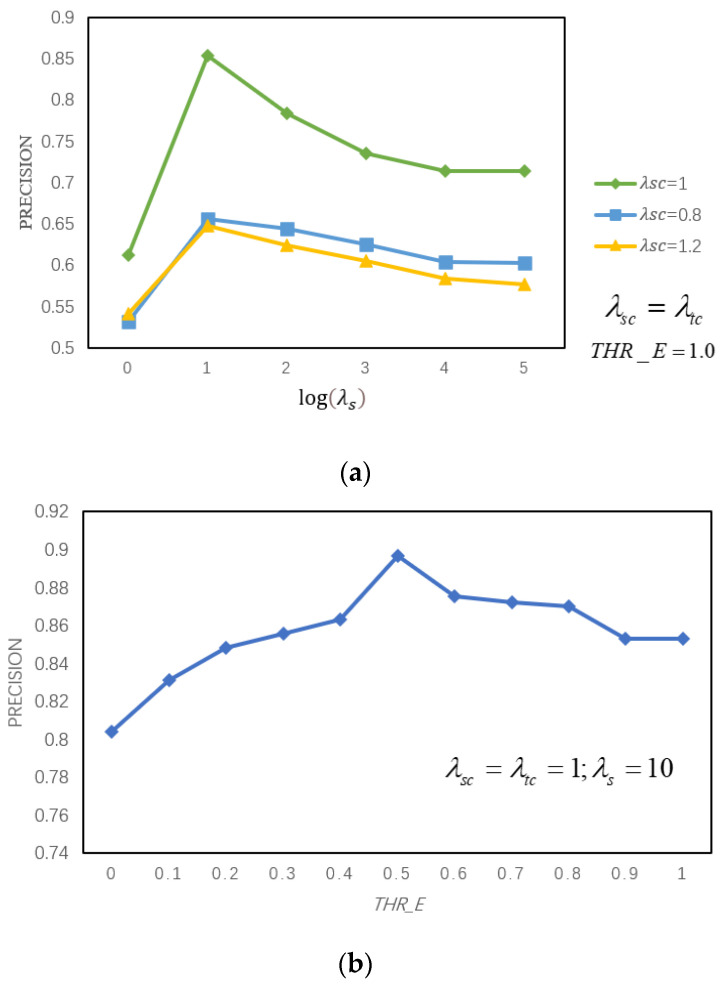
Ablation study w.r.t. hyperparameters $\lambda_{sc}\Delta \lambda_{tc},\lambda_{s}$ and *THR_E*, (**a**) when $\lambda_{sc}=\lambda_{tc}$; THR_E=1.0, the precision with the variation of $\lambda_{sc}$ (**b**)when $\lambda_{sc}=\lambda_{tc}=1;\;\lambda_{s}=10$, the precision with the variation of *THR_E*.

We download 150 videos with noticeable brightness changes from the video website and adopt the metrics of ACE forensics [35] to determine the brightness changes of videos. We found that these videos have a higher intensity of dynamic brightness changes than the experimental video library. Because the authenticity of the website video is uncertain, it cannot be used as an experimental video. Therefore, we apply the model of dynamic brightness change, which is shown in Equation (21), to simulate the video brightness changes in the real-life environment. We report the precision with respect to $\lambda_{sc},\;\lambda_{tc},\lambda_{s}$ and *THR_E* respectively. Based on the results of Figure 6, we observe the effect is best when $\lambda_{sc}=1,\;\lambda_{tc}=1,\;\lambda_{s}=10$ and $THR_{E}=0.5$. The values of THR_R and THR_R1 are set according to the Chebyshev inequality adaptively [36], and the corner point c(i) in Algorithm 1 is set to 50.

To evaluate the performance of the detection algorithm, we use the error metrics of precision and recall to analyze the experimental results. The calculation Equations are:

(24) $$precision=\frac{N_{c}}{N_{c}+N_{f}}$$

(25) $$recall=\frac{N_{c}}{N_{c}+N_{m}}$$

where $N_{c}$ is the number of detected correct points, $N_{f}$ is the number of detected false points, and $N_{m}$ is the number of tampered points that were missed.

### 6.3. Experimental Results

**Figure 7:**
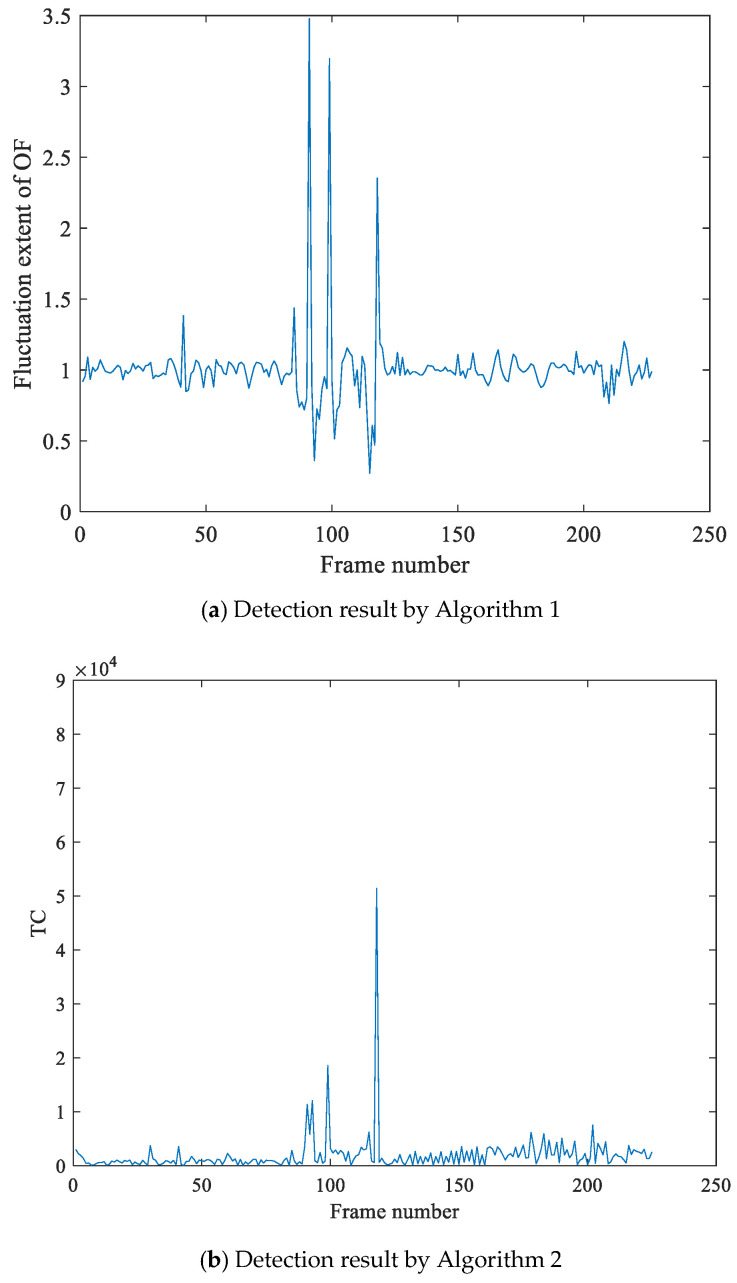
Detection result of frame deletion forgery for the video with jitter noises and illumination noises. In (**a**), Algorithm 1 utilizes the fluctuation extent of OF to detect forgery, and the detection results show that the feature sequence has peaks pair (91, 99, 118). At the same time, the motion entropy of OF is greater than the selected threshold, which indicates it is a jittery video. Therefore, we detect the video by Algorithm 2, and the detection results in the (**b**) show that frame118 is the tampering point.

Figure 7 is the detection result of frame deletion forgery for the video with jitter noises and illumination noises. Figure 7a is the experimental results by Algorithm 1, which shows the OF fluctuation feature sequence has peaks pair (91, 99, 118). At the same time, the calculated value of motion entropy ME is 0.672, which indicates that the video is jittery. To reduce the side effect of the video jitter, we detect the nervous video by Algorithm 2, which utilizes the texture changes fraction feature TC to detect. The detection result of double-checking is shown as Figure 7b, where the tampering point is 118. At last, we make the judgment of video tamper by Algorithm 3, We can obtain that 118 is frame deletion forgery point, and the peak pair (91, 99) is false detection results.

**Figure 8:**
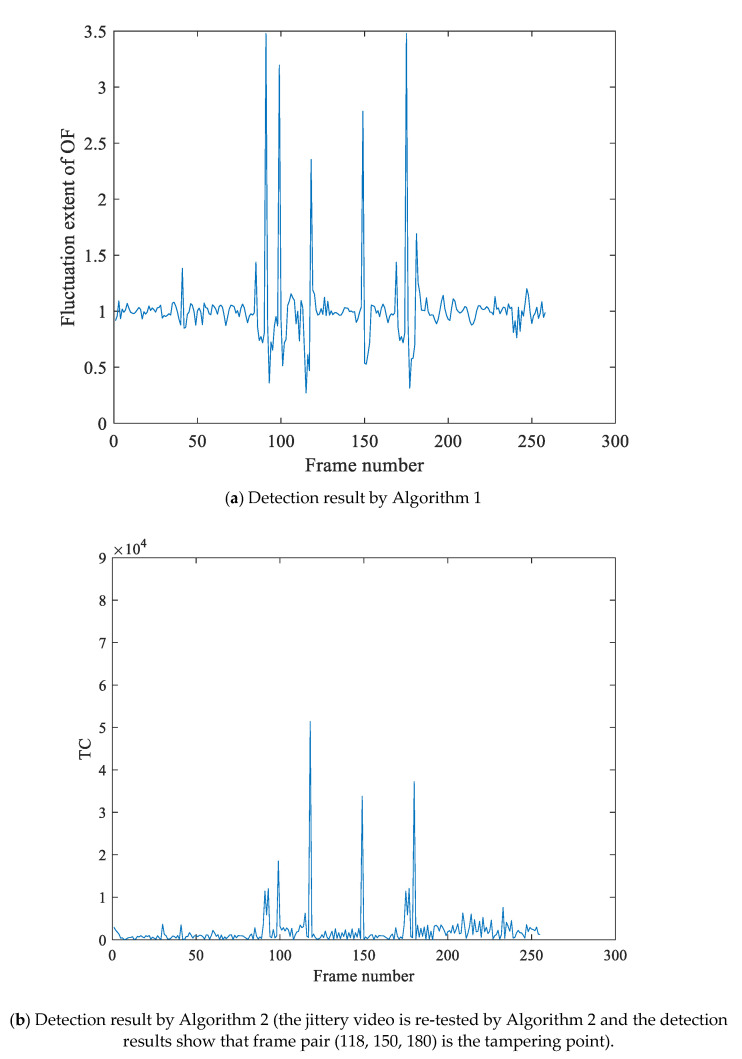
The multi-tamper detection result of jitter video with jitter noises and illumination noises. In (**a**), Algorithm 1 utilized the fluctuation extent of OF to detect forgery, and the detection results show that the feature sequence has peaks pair (91, 99, 118, 150, 180). At the same time, the motion entropy of OF is greater than the selected threshold, which indicates the video is a jittery video; this video is re-tested using Algorithm 2, and the detection results in (**b**) show that frame pair (118,150,180) is the tampering point.

Based on the detection result of Figure 7, Figure 8 is the detection result of multiple tampering of the same video. Figure 8a is the experimental results by the Algorithm 1, which shows that the OF fluctuation feature sequence has peaks pair (91, 99, 118, 150, 180). Moreover, the motion entropy ME is 0.752, which indicates that the video is jittery. To eliminate the effect of the video jitter, this video is re-tested by Algorithm 2, The re-testing detection result is shown as Figure 8, which locates the tampering points pair at (118, 150, 180), and the peak pair (91, 99) is false detection results. At last, we make the judgment of tamper by Algorithm 3. We can obtain that frame118 is the deletion forgery point, and the point pair (150, 180) is frame insertion forgery point.

**Figure 9:**
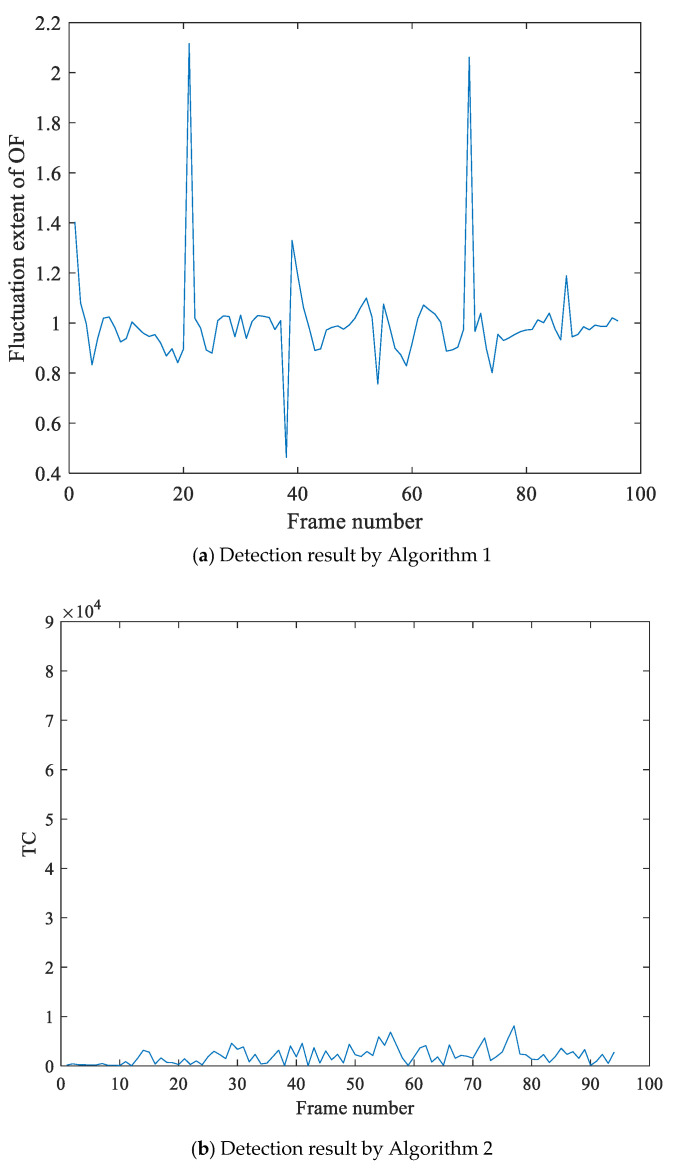
Detection result of the untampered video with jitter noises and illumination noises. In (**a**), Algorithm 1 utilizes the fluctuation extent of OF to detect forgery, and the detection results show that the feature sequence has peaks pair (22, 70). At the same time, the motion entropy of OF is greater than the selected threshold, which indicates the video is jittery; this video is re-tested using Algorithm 2 and it can be seen that the texture changes fraction sequence has no peaks in (**b**).

Figure 9 is the detection result of the untampered video with jitter noises and illumination noises. Figure 9a is the detection result by Algorithm 1, which shown that the OF fluctuation feature sequence has a peak pair (22, 70). The motion entropy ME is 0.643, which indicates the video is jittery. To eliminate the effect of the video jitter, this video is re-tested by the Algorithm 2, which utilizes the texture changes fraction to detect. The detection result of re-testing is shown as Figure 9b, which indicates that the texture changes fraction sequence has no peaks. Based on the above test results, we judge that the video is original and has not been tampered.

**Figure 10:**
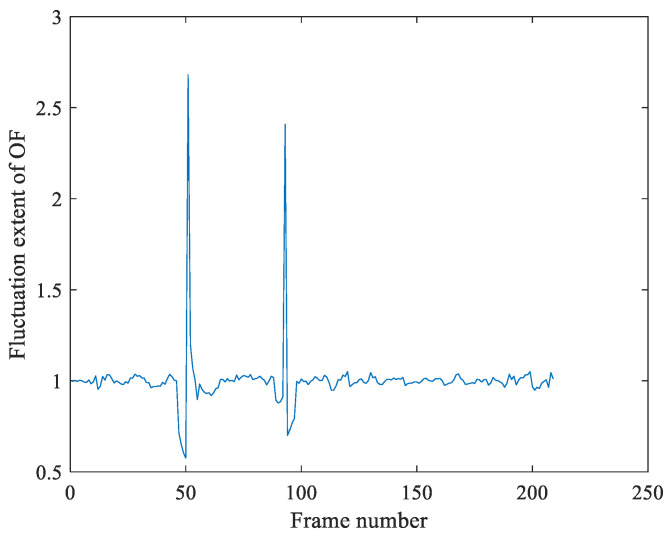
Detection result of frame replacement forgery of video with illumination noise.

Figure 10 shows frame replacement forgery detection result of video with illumination noise. It shows that the feature sequence has peaks pair (51, 93). At the same time, the calculated motion entropy ME is 0.453, which indicates that the video is not jittery. Then we judge video tamper, and the OF fluctuation feature *r* between frame 50th and 94th is 1.0046, which shows frame pair (50, 94) is very similar. Therefore, the peak pair (51, 93) is the location of video insertion forgery.

**Figure 11:**
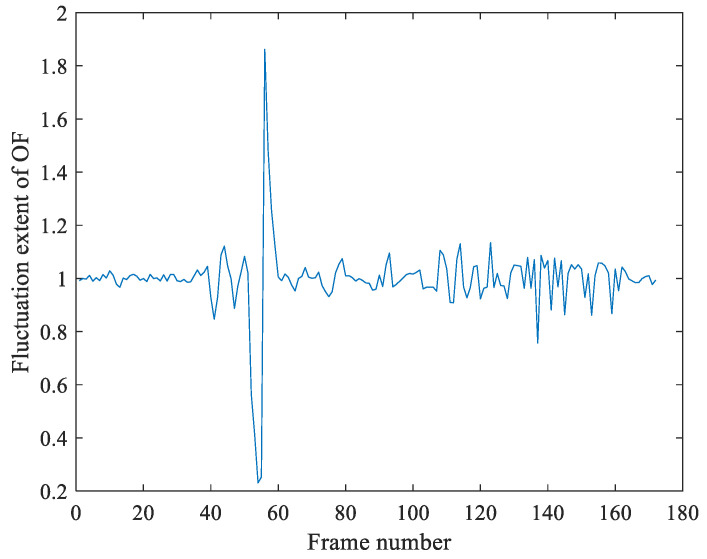
Detection result of frame deletion forgery of video with illumination noise.

Figure 11 shows the detection result of frame deletion forgery of video with illumination noises. It indicates the OF fluctuation feature *r* has prominent peaks at frame deletion point 56. Because the motion entropy ME is 0.486, which suggests that the video is not jittery. At last, we make the judgment of video tamper and obtain that frame point 56 is the location of video deletion forgery.

**Figure 12:**
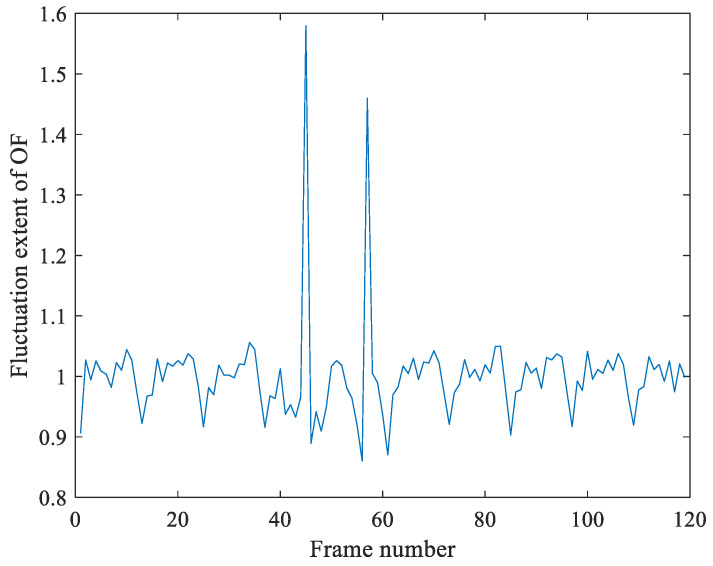
Detection result of frame copy-move forgery of video with illumination noise.

Figure 12 is the detection result of video frame copy-move forgery of video with illumination noise. Figure 12 is the detection result by Algorithm 1, which shown that the OF fluctuation feature sequence has a peak pair (45, 57). And we calculate the value of motion entropy ME is 0.482, which indicates that the video is not jittery. At last, we make the judgment of video tamper. The OF fluctuation feature *r* between frame 44th and 58th is 0.9844. Therefore, the peak pair (45, 57) is the location of video insertion forgery.

According to the performance evaluation criteria of the proposed algorithm, a comparison is made between the proposed algorithm in the paper and the state-of-the-art different video tamper detection algorithms [3,6,9,37]. Table 3 shows the parameter description of the comparison methods, our proposed method and the comparison methods use the same dataset, and the comparison results are shown in Table 4.

As compared to methods reported in [3,6,9,37], the proposed method has high robustness and high accuracy. The results indicate that the proposed method is capable of effective detection and localization of all inter-frame forgeries on videos with illumination noises and jitter noises. In a real-life scenario, the forensic investigator has no control over the parameters of the environment where the video was captured or the parameters used by the video tamper. The forensic investigator must detect in the complete absence of any information regarding the noises, the motion-level, and the forgery operation forms of the captured video. Therefore, the most suitable forgery detection is the one that has practical suitability for the real-life video scenes, such as videos with brightness variance, videos with significant jitter, and the various motion-level videos. Furthermore, our method not only can locate the forgery precisely, but also can estimate the way of multi-forgery on tampered positions.

For [3,6], the detection methods based on OF are invalid when there are illumination changes added to the image sequence. Hence, the detection result is not so good. For [9], the detection performance is improved; the main reason is that the Zernike moment feature avoids the effect of brightness intensity. However, experiments prove that its detection performance on the jittery video has decreased significantly, so the detection result is not so good. For [37], the test results are also relatively improved; the main reason is that the multi-channel feature avoids missing detection; however, experimental results show that the performance of this method is not good for the minor frame deletion forgery, so this method is not as stable as the proposed method in our paper.

Prior video tampering detection methods are not suitable for videos with dynamic brightness changes and jittery videos. The detection method [13] based on motion residual can be ideal for the most motion-level video, such as high motion-level, medium motion-level, etc. However, it is not suitable for the slowest motion-level video. The inter-frame difference will decrease as the video motion-level decrease, so the extracted motion residual feature will be weak. However, the relocated I-frame is not affected by the motion level of video, so the relocated I-frame will be defined as the tampered frame mistakenly. Therefore, reference [13] is not suitable for the lowest motion video. Our proposed method utilizes the inconsistencies of features, including the enhanced OF  and texture changes fraction, to detect tamper in real-life videos. The former feature is insensitive to the motion level of the video. Moreover, the latter feature can also describe the subtle inter-frame differences of the lowest motion video. Therefore, our method is also suitable for the lowest motion video.

To reduce the effect of illumination noises and jitter noises, we utilize a robust optical flow detection method based on relaxing brightness consistency assumption and intensity normalization, which can reduce the influence of significant brightness change and small brightness change, respectively. At the same time, we use motion entropy ME to sense whether the video is jittery and utilize the texture changes fraction TC for double-checking, so the false detection caused by video jitter can be reduced. Experiments prove that the proposed detection method has strong robustness and high accuracy for complex scene video.

## 7. Conclusions

In this paper, we have proposed a novel detection framework for inter-frame forgery in real-life video with illumination noises and jitter noises. Firstly, for videos with severe brightness changes, we relax brightness constancy constraint and adopt intensity normalization to propose a new optical flow algorithm in Algorithm 1. Secondly, for videos with large jitter noises, we introduce motion entropy to detect the jitter and extract the stable feature of texture changes fraction for double-checking in Algorithm 2. Finally, we make the judgment of video tamper in Algorithm 3. The proposed method was validated by extensive experimentation in diverse and realistic forensic setups. The obtained results indicate that the proposed method is entirely accurate and robust. It can detect video single-forgery or multi-forgeries with an average accuracy of 89%, including frame deletion, frame insertion, frame replacement, and frame copy-move. Furthermore, the proposed method is not sensitive to the jitter noises, illumination noises, or the motion level of the video. In the future, it would be beneficial to explore the suitability of some other real-life video scenes, such as blurred video and still video.

## Figures and Tables

**Table 1:** Description of different approaches and parameter settings used for OF evaluation on Hydrangea images with brightness change.

Approaches	Descriptions and Parameter Settings
HS	Classical H&S method, λ=1000.
HS + IN	H&S method with Intensity Normalization, λ=1000.
HS + BR	H&S method with Brightness Relaxing factor, $\lambda_{s}=10,\lambda_{sc}=1,\lambda_{tc}=1$, and d = 0.35.
the enhanced OF algorithm	combine HS+BR and intensity normalization.

**Table 2:** Error measures of different OF methods between frame10 and frame11 of Hydrangea images under brightness change.

Approaches	AAE	Average EPE	Time (s)
HS	13.188	1.350	6.07
HS + IN	7.074	0.776	6.62
HS + BR	28.497	6.631	7.08
Enhanced OF algorithm	4.175	0.389	8.06

**Table 3:** Parameter description of comparison methods.

Parameters	Methods				
	Ref. [3]	Ref. [6]	Ref. [9]	Ref. [37]	Proposed
Consider the illumination noise	No	No	Not validated	Not validated	Yes
Consider the jitter noise	Not validated	Not validated	Not validated	Not validated	Yes
Validation by multi-forgery	No	No	No	No	Yes
Forgery detected	Removal/Insertion/copy-move	Copy-move	Removal/insertion/copy-move	Removal/insertion/copy-move	Removal/insertion/copy-move

**Table 4:** Comparison with state-of-the-art algorithms.

Method	Precision	Recall
Proposed	0.8968	0.8952
Ref. [3]	0.5134	0.5142
Ref. [6]	0.5262	0.5193
Ref. [9]	0.6321	0.6352
Ref. [37]	0.6454	0.6336

## Data Availability

Data sharing not applicable.
